# *Aeromonas sobria* Induces Proinflammatory Cytokines Production in Mouse Macrophages *via* Activating NLRP3 Inflammasome Signaling Pathways

**DOI:** 10.3389/fcimb.2021.691445

**Published:** 2021-08-26

**Authors:** Wei Zhang, Zhixing Li, Haitao Yang, Guanglu Wang, Gang Liu, Yu Wang, Babatunde Kazeem Bello, Panpan Zhao, Wei Liang, Jingquan Dong

**Affiliations:** ^1^Key Jiangsu Institute of Marine Resources Development, Jiangsu Key Laboratory of Marine Pharmaceutical Compound Screening, Jiangsu Ocean University, Lianyungang, China; ^2^State key laboratory of Rice Biology, Lianyungang Academy of Agricultural Sciences, Lianyungang, China; ^3^Laboratory Department of Ningbo First Hospital, Ningbo Hospital of Zhejiang University, Ningbo, China

**Keywords:** *Aeromonas sobria*, NLRP3 inflammasome, caspase-1 p20, proinflammatory cytokines, immune response

## Abstract

*Aeromonas sobria*, a common conditional pathogenic bacteria, is widely distributed in the environment and causes gastroenteritis in humans or septicemia in fish. Of all *Aeromonas* species, *A. sobria* is the most frequently isolated from human infections especially in immunocompromised subjects. Innate immunity is the first protection system of organism to resist non-specific pathogens invasion; however, the immune response process of hosts against *A. sobria* infection re\mains unexplored. The present study established an *A. sobria* infection model using primary mouse peritoneal macrophages (PMφs). The adherence and cytotoxicity of *A. sobria* on PMφs were determined by May-Grünwald Giemsa staining and LDH release measurement. Pro-inflammatory cytokine expression levels were measured using qPCR, western blotting, and ELISA methods. We also investigated the levels of ASC oligomerization and determined the roles of active caspase-1 in IL-1β secretion through inhibition assays and explored the activated pattern recognition receptors through immunofluorescence. We further elucidated the roles of activated inflammasome in regulating the host’s inflammatory response through inhibition combined with ELISA assays. Our results showed that *A. sobria* induced lytic cell death and LDH release, whereas it had no adhesive properties on PMφs. *A. sobria* triggered various proinflammatory cytokine transcription level upregulation, and IL-1β occupied the highest levels. The pro-IL-1β protein expression levels increased in a dose-dependent manner with MOI ranging from 1 to 100. This process was regulated by ASC-dependent inflammasome, which cleavage pro-IL-1β into active IL-1β p17 with activated caspase-1 p20. Meanwhile, the expression levels of NLRP3 receptor significantly increased, location analysis revealed puncta-like surrounding nuclear, and inhibition of NLRP3 inflammasome downregulated caspase-1 activation and IL-1β secretion. Blocking of NLRP3 inflammasome activation through K^+^ efflux and cathepsin B or caspase approaches downregulated *A. sobria*–induced proinflammatory cytokine production. Overall, these data indicated that *A. sobria* induced proinflammatory cytokine production in PMφs through activating NLRP3 inflammasome signaling pathways.

## Introduction

*Aeromonas* species are the causative agents of varieties of illnesses, including gastrointestinal infections and extraintestinal localizations in open wounds, hepatobiliary system, or eyes (King et al., 1992). Among the *Aeromonas* isolates from human infections, a number of 85% cases are related to *Aeromonas hydrophila*, *Aeromonas caviae*, and *Aeromonas sobria* (*A. sobria*) (Janda and Abbott, 1998); and *A. sobria* is more frequently associated with human infections (Daily et al., 1981). *A. sobria* is a Gram-negative, single-flagella, rod-shaped, motile, facultative anaerobic bacterium of genus *Aeromonas* (Taslimi et al., 2018). It is widely distributed in the natural environments, including water, soil, feces, etc., being a conditional pathogenic bacterium to humans, aquatic animals, livestock, and poultry (Cahill, 1990; Song et al., 2019). Humans or animals get infected through ingestion of contaminated food or direct contact, exhibiting symptoms of bacteremia, tissue damage, pneumonia, meningitis, gastroenteritis, or septicemia, especially in people with weakened immune system (San Joaquin and Pickett, 1988; Su et al., 2013; Wang et al., 2009). Bacterial disease resistant to antibiotics is a huge challenge for the existence of a plasmid encoding β-lactamase in *A. sobria* (Stano et al., 2008; Lim and Hong, 2020). Thus, deep research in the interaction between *A. sobria* and hosts is urgently necessary to develop effective strategies against *A. sobria* infection.

Innate immunity is the first protection system of organism to resist non-specific pathogen invasion through recognizing a vast array of pathogen-associated molecular patterns (PAMPs) or danger-associated molecular patterns (DAMPs) released by microorganisms during infection. Pattern recognition receptors (PRRs) in the innate immune system and their mediated downstream signaling pathways, such as cytosolic nucleotide-binding oligomerization (NOD)-like receptors (NLRs), membrane-associated Toll-like receptors (TLRs), absent in melanoma 2 (AIM2)-like receptors, etc., have been widely studied (Thaiss et al., 2016). Activation of TLRs or NLRs lead to upregulation of numerous genes’ transcription, such as cytokines of pro-IL-1β, pro-IL-18 (Chen et al., 2009; Fitzgerald and Kagan, 2020). Among them, NLR family, pyrin domain containing 3 (NLRP3) is a kind of well-studied intracellular innate immune receptor and can be activated by various bacterial strains, viruses, non-infectious crystals, K^+^ efflux, reactive oxygen species generation, as well as pore-forming toxins (Muñoz-Planillo et al., 2013; He et al., 2016; Platnich et al., 2018; Zhao and Zhao, 2020). Upon activation, it recruits apoptosis-associated speck-like protein (ASC) and pro-caspase-1 to form an inflammasome complex, which generates active caspase-1 and cleavage pro-IL-1β or pro-IL-18 into mature IL-1β or IL-18 and thus initiates inflammation and regulates the interaction between host-microbes (He et al., 2016). Previous studies showed that *Aeromonas veronii* induced mouse macrophage apoptosis accompanied with caspase-1 activation *via* NLRP3 inflammasome (McCoy et al., 2010a). *Aeromonas hydrophila* triggered host proinflammatory responses through activation of caspase-1 and release of IL-1β (McCoy et al., 2010b). *A. sobria* is the most important member of genus *Aeromonas* with a mortality rate of 56% when it causes bacteremia (Martins et al., 2002). The roles of NLRP3-caspase-1-IL-1β axis in *A. sobria* infection have not been clarified.

The present study established an *A. sobria* infection model using primary mouse peritoneal macrophages and verified that *A. sobria* induced inflammatory response through activation of caspase-1 and formation of NLRP3 inflammasome complex, and inhibition of NLRP3 inflammasome-mediated IL-1β p17 secretion downregulated *A. sobria* mediated inflammatory response *in vitro*. Deep research in the mechanisms of *A. sobria*–induced inflammation will provide new targets for the treatment of *A. sobria* infection.

## Materials and Methods

### Bacterial Strains and Mice

*A. sobria* strain ATCC 43979 was purchased from American Type Culture Collection (ATCC, Manassas, VA, USA) and cultured at 30°C in nutritive broth medium [10 g/L peptone, 5 g/L NaCl, 3 g/L beef extract (Sinopharm, Beijing), pH 7.2 ± 0.2] for 36 h. Six eight-week-old SPF level C57BL/6 female mice were purchased from Pizhou Oriental Breeding Company, housed in filter-top cages under conditions of 12 h light/dark cycles, fed with sterile drinking water, and SPF-level mouse feed *ad libitum*. All mice experiments have been approved by the research ethics from the Animal Welfare and Research Ethics Committee of Jiangsu Ocean University (Permit Number: 2017124242).

### Isolation and Culture of Primary Mouse Peritoneal Macrophages (PMφs)

Mice were injected (i.p.) with 2.5 ml of sterile Difo Fluid thioglycollate medium (BD, USA). After 3–4 d, mice were euthanized through cervical dislocation, and the enriched macrophages in the peritoneal cavity were harvested. After centrifugation at 1,000 × g for 10 min, the PMφs were resuspended in RPMI 1640 medium supplemented with 10% fetal bovine serum (Biological Industries, Israel) and cultured in six-well plates (ThermoFisher, USA) at a density of 4.5 × 10^6^ cells/well at 37°C and 5% CO_2_. After sedimentation for 6 h, non-adherent cells were removed and incubated for 12 h prior to stimulation (Zhao et al., 2021).

### Cytotoxicity Assays

Cytotoxicity assays in PMφs were evaluated by measuring the levels of lactate dehydrogenase (LDH) release. The PMφs were seeded in 96-well plates at a density of 1 × 10^5^ cells/well, stimulated with *A. sobria* (MOI=1, 10, 100) for 90 min and incubated at 37°C for 12 h. The culture medium was collected and centrifuged at 12,000 × for 1 min under 4°C. After removal of the cell pellets, the supernatants were used for LDH level measurement using LDH Cytotoxicity Test Kit (Beyotime, Beijing) according to manufacturer’s instructions (Wang et al., 2017; Wu et al., 2019).

### Adhesion Assays

Adhesion capacity was tested by stimulating PMφs with *A. sobria* (MOI=1, 10, 100) for 90 min. Then, cells were washed three times with sterile PBS, fixed with 2% formaldehyde, stained with May-Grünwald Giemsa, observed under optical microscope, and expressed as the percentage of cells with more than 10 adherent bacteria on the cell surface (Stano et al., 2008; Dallagassa et al., 2018).

### Real-Time Quantitative PCR (qPCR)

Total RNA was extracted from *A. sobria*–infected PMφs pellets [multiplicities of infection (MOI) = 10] using TRIzol reagent (Monad, Wuhan) according to the manufacturer’s instructions. Two micrograms of RNA was first incubated with dsDNase (Thermo Scientific, USA) to remove genomic DNA and then reverse transcribed into cDNA with MonScript RTIII Super Mix (Monad, Wuhan). The single-strand cDNA was diluted 20 times and amplified with MonAmp SYBR Green qPCR Mix (High ROX, Monad, Wuhan) on an ABI StepOne Plus real-time PCR machine. The reaction system and procedure were set according to the manufacturer’s manual (Zhao et al., 2021). Results were analyzed through melting curves, and mRNA fold change was calculated using 2^−ΔΔCt^ method. Primer sequences are listed in **Tables 1** and **2**.

**Table 1:** qPCR primer sequences of inflammatory cytokines.

Name	GenBank number	Primer sequence (5’to 3’)
IL-1β	NM_008361	F: AGGAGAACCAAGCAACGACA
		R: CTCTGCTTGTGAGGTGCTGA
IL-6	NC_000071	F: TGCCTTCTTGGGACTGATGC
		R: GCAAGTGCATCATCGTTGTTC
IL-10	NC_000067	F: GCAGTGGAGCAGGTGAAGAG
		R: CGGAGAGAGGTACAAACGAGG
IL-12	MMU23922	F: TACAAGGTTCAGGTGCGAGC
		R: ATGTATCCGAGACTGCCCAC
IL-18	NM_008360	F: ACCAAGTTCTCTTCGTTGAC
		R: CTTCACAGAGAGGGTCACAG
TNF-α	NM_013693	F: GACGTGGAACTGGCAGAAGA
		R: GGCTACAGGCTTGTCACTCG
IFN-γ	NM_008337	F: CGGCACAGTCATTGAAAGCC
		R: TGTTGTTGCTGATGGCCTGA
Actb	NM_007393	F: GCCATGTACGTAGCCATCCA
		R: ACGCACGATTTCCCTCTCAG

F represented forward primer.

R represented reverse primer.

**Table 2:** qPCR primer sequences of PRRs.

Name	GenBank number	Primer sequence (5’to 3’)
NOD1	NM_172729.3	F: GATTGGAGACGAAGGGGCAA
		R: CGTCTGGTTCACTCTCAGCA
NOD2	NM_145857.2	F: GCCAGTACGAGTGTGAGGAG
		R: GCGAGACTGAGTCAACACCA
NLRP3	NM_145827.4	F: AGCCAGAGTGGAATGACACG
		R: CGTGTAGCGACTGTTGAGGT
NLRC4	NM_001033367	F: GCTCAGTCCTCAGAACCTGC
		R: ACCCAAGCTGTCAATCAGACC
NLRC5	NM_001033207	F: TCTCTAAGCAGCTAGGGGCA
		R: GGGGAGTGAGGAGTAAGCCA
AIM2	NC_000067	F: ACTTTCCTGGAGCACGGGAT
		R: ATGCCCTAGTTTTACCCACTCC

### Western Blotting

The *A. sobria*–infected PMφs pellets (MOI=1, 10, 100) were lysed in RIPA buffer containing 1 mM phenylmethylsulfonylfluoride (PMSF, Solarbio, Beijing). The lysed PMφ pellets and *A. sobria*–infected PMφ supernatants were concentrated using methanol and chloroform as previously described (Wang et al., 2018a) and quantified using BCA method with BCA Protein Assay Kit (Thermo Scientific, USA). Equal amounts of protein extraction (30 μg) were separated by SDS-PAGE electrophoresis and transferred onto PVDF membrane (Millipore, USA). The protein-contained membranes were blocked in 5% non-fat milk; incubated at 4°C overnight with primary antibodies, including IL-1β (1:2,000, R&D, USA), caspase-1 (p20) (1:1,000, Adipogen, Switzerland), NLRP3 (1:1,000, Adipogen, Switzerland), ASC (1:500, Wanleibio, Shenyang), and β-actin (1:5000, Proteintech, Wuhan); incubated at room temperature for 1 h with horseradish peroxidase-conjugated secondary detection antibodies, including rabbit anti-goat IgG, goat anti-mouse IgG (H+L), and goat anti-rabbit IgG (1:2,000, Proteintech, Wuhan); and visualized with Immobilon Western Chemiluminescent HRP substrate (Millipore, USA) on a ChemiScope Western Blot Imaging System (Clinx, Shanghai).

### Enzyme-Linked Immunosorbent Assays (ELISA)

The *A. sobria*–infected PMφs supernatants (MOI=1, 10, 100) were harvested and centrifuged at 12,000 × for 1 min under 4°C. After removal of the pellets, the supernatants were used for cytokines of IL-1β, IL-6, IL-12, and TNF-α measurement using mouse-specific ELISA kits (Invitrogen, USA) according to the manufacturer’s instructions. The optical density (OD) at 450 nm value of each well was read on a microplate reader (Biotek, USA), and the protein concentration was calculated according to the standard sample-generated standard curves.

### Immunofluorescence Staining

The PMφ were cultured on sterile glass coverslips and stimulated with *A. sobria* at a MOI of 10 for 6 h. The cells-covered glass coverslips were fixed in 4% paraformaldehyde (Biosharp, Beijing) at room temperature for 10 min; permeabilized in 0.1% Triton X-100 at room temperature for 20 min; blocked in 5% BSA at room temperature for 2 h; incubated with 200 times diluted NLRP3 antibodies at 4°C overnight and FITC-conjugated rabbit anti-goat IgG (H+L) (1:400, Earthox, USA) at 37°C for 1 h; stained with nuclei dye of 4’, 6-diamidino-2-phenylindole, dihydrochloride (DAPI, 1 μg/ml, Thermo Scientific, USA); and visualized on a fluorescence microscope (Olympus, Japan). The fluorescence intensity of NLRP3 was measured using Fiji software, and the parameters were set as follows: Image type 8-bit, default threshold, analyze measurements of area and integrated density. The mean fluorescence intensity of NLRP3 was calculated using formula of (integrated density/area), and graph was generated from two independent assays.

### Inhibition Assays

PMφ was pretreated with either 100 μM Ac-YVAD-CHO (an inhibitor of caspase-1 and -4; Enzo Life Science, Switzerland) or 10 μM zVAD-fmk (an inhibitor of pan-caspase; Selleck, Shanghai) for 1 h before *A. sobria* stimulation (MOI=10) to explore the roles of caspase-1 p20 in activated IL-1β secretion (Malyshev et al., 2004; Cao et al., 2005). PMφs were pretreated with either 25 μM CA-074 methyl ester (CA-074 Me, an inhibitor of cathepsin B, MedChemExpress, USA) or 50 μM Glyburide (an inhibitor of NLRP3 by inhibiting K^+^ efflux; Selleck, Shanghai) for 1 h before stimulation to block NLRP3 receptor activation and explore its roles in caspase-1 p20 production and IL-1β p17 secretion (Lamkanfi and Dixit, 2009; Liu et al., 2013). PMφs were pretreated with these NLRP3 inflammasome inhibitors through different ways to detect its roles in inflammatory response by measuring cytokines levels of IL-1β, IL-6, IL-12, and TNF-α.

### ASC Oligomerization

ASC oligomerization assays were carried out as previously described with a little modification to the protocol (Wang et al., 2018b). The *A. sobria*–infected PMφs pellets (MOI=10) were washed three times with cold PBS and then disrupted using a 27-gauge needle (Millipore, Billerica, MA, USA) in lysate buffer [25 mM Na_2_PO_4_ sodium phosphate, 187.5 mM NaCl, 25 mM HEPES, 125 mM NaHCO_3_ (Sinopharm, Beijing)]. The suspensions were centrifuged at 5,000 × g for 3 min. After removal of the supernatants, the pellets were dissolved in 40 µl of lysate buffer and cross-linked with 10 µl of 2 mM fresh DSS (Aladdin, Shanghai) at 37°C for 30 min. Then, the cross-linked mixture was centrifuged at 5,000 × g for 10 min under 4°C, and the supernatants were stopped by mixing with SDS loading buffer (TransGen, Beijing) at room temperature for 15 min and then used for western blotting analysis.

### Statistical Analysis

Data of mean ± Standard Deviation were from three independent assays with three technical repeats. Differences between two groups were analyzed using a Student’s *t*-test, and three or more groups were analyzed using one-way analysis of variance (ANOVA). Graphs were generated by GraphPad Prism 8.00 software (Inc., La Jolla, USA). P-values of less than 0.05, 0.01, or 0.001 were all regarded as statistically significant and described as “*,” “**,” or “***,” whereas p-values of more than 0.05 were regarded as not statistically significant and displayed as “n.s.”

## Results

### *A. sobria* Triggers PMφ Death and Induces Inflammatory Response Through Upregulation of Various Pro-Inflammatory Cytokines

**Figure 1:**
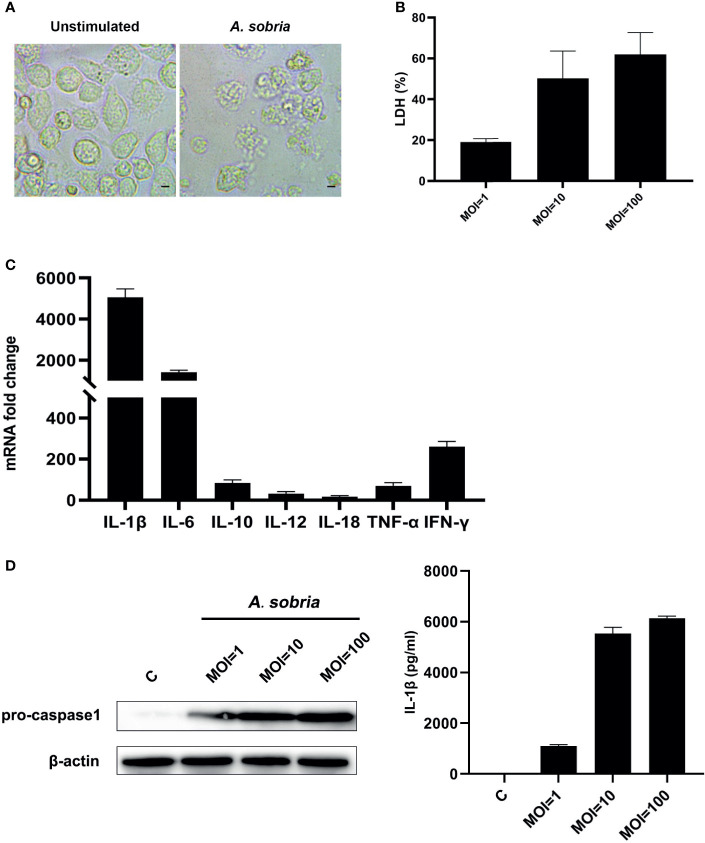
*A sobria* triggered PMφs death and inflammatory response. PMφs were infected with *A sobria* (MOI = 1, 10, 100) for 90 min, and then *A sobria* was discarded. After washing three times with sterile PBS, PMφs were treated with gentamicin sulfate (100 μg/ml) containing RPMI 1640 maintenance medium for 2 h to kill residual *A sobria* and then replaced with gentamicin sulfate (20 μg/ml) containing RPMI 1640 maintenance medium for 12 h **(A)** Phase contrast images of unstimulated or *A sobria*–stimulated (MOI = 10) PMφs. Bar = 10 μm. **(B)** The cytotoxicity was evaluated by measuring the LDH release from *A sobria*–infected supernatants (MOI = 1, 10, 100). **(C)** RNA was extracted from infected cells (MOI = 10), and cDNA was synthesized for pro-inflammatory gene transcription levels analysis using qPCR. **(D)** Cells were lysed from infected cells (MOI = 0, 1, 10, 100), and protein was extracted for pro-IL-1β expression analysis using western blotting. Meanwhile, supernatants were harvested, and IL-1β protein levels were measured using ELISA. Data were presented as mean ± Standard Deviation from three independent assays.

The *A. sobria*–stimulated PMφs underwent lytic cell death in comparison with the negative control group (**Figure 1A**). The *A. sobria* did not show adhesive properties on PMφs; however, it induced LDH release in a dose-dependent manner. The mean levels of LDH release in the *A. sobria*–stimulated supernatants were 19.01, 50.13, and 61.87% under MOI=1, 10, 100 (**Figure 1B**). To determine the function of *A. sobria* in PMφs, many pro-inflammatory cytokines were determined after 90 min of infection with *A. sobria* at an MOI of 10 and 2 h gentamycin treatment followed by 12 h incubation. As shown in **Figure 1C** and **Figure S1**, the mRNA fold changes of IL-1β, IL-6, IL-10, IL-12, IL-18, TNF-α, and IFN-γ were upregulated to different levels and IL-1β increased to the highest level. To further verify the protein expression levels of IL-1β, western blotting assays were conducted after 12 h incubation at a MOI of 1, 10, and 100, respectively. Results showed that *A. sobria* incubation induced pro-IL-1β protein expression, and the upregulation levels were in a dose-dependent manner. Meanwhile, the IL-1β secretion levels were also evaluated using ELISA assays, and the results revealed that *A. sobria* induced low levels of IL-1β secretion at MOI=1 and increased remarkably when stimulation at MOI=10 or 100 (**Figure 1D**).

### *A. sobria*–Mediated IL-1β p17 Secretion Is Regulated by ASC-Dependent Inflammasome

**Figure 2:**
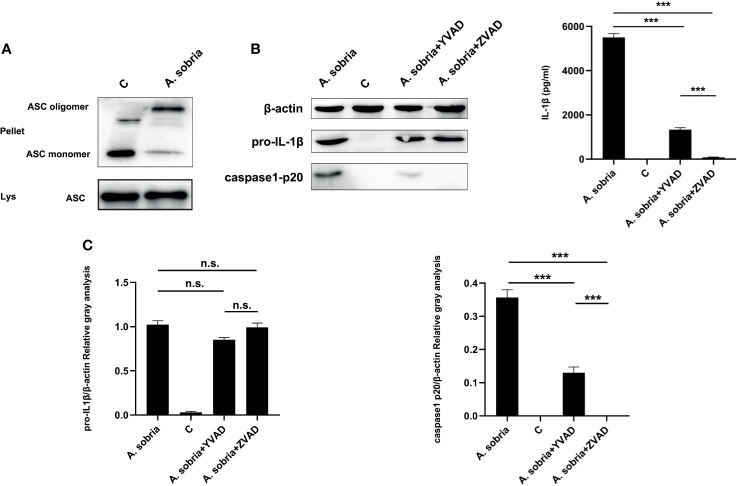
*A sobria*–induced IL-1β secretion was regulated by ASC-dependent inflammasome activation. PMφs (4.5 × 10^6^ cells/well in six-well plate) were pretreated with either Ac-YVAD-CHO (100 μM) or zVAD-fmk (10 μM) for 1 h, infected with *A sobria* (MOI= 10) for 90 min, and then *A sobria* was discarded. After washing three times with sterile PBS, PMφs were treated with gentamicin sulfate (100 μg/ml) containing RPMI 1640 maintenance medium for 2 h to kill residual *A sobria* and then replaced with gentamicin sulfate (20 μg/ml) containing RPMI 1640 maintenance medium for 12 h The activated caspase-1 p20 was detected in the supernatants using western blotting, and mature IL-1β p17 was detected in the supernatants using ELISA. No inhibitor treatment group was used as positive control group, and no *A sobria* inoculation group was used as negative control (C) group. **(A)** The ASC oligomerization levels were evaluated in the *A sobria* inoculation group and C group. Pellet represented cross-linked cell pellet, and Lys represented lysed cells. **(B)** The protein expression of caspase-1 p20, pro-IL-1β, and mature IL-1β was detected in the inhibitor pretreatment groups, *A sobria* inoculation group, and C group. **(C)** The relative gray values of caspase-1 p20 and pro-IL-1β were normalized to β-actin through Image. Data were presented as mean ± Standard Deviation from three independent assays with three technical repeats. Significant difference was analyzed using SPSS software, and graph was generated using GraphPad Prism 8 software. *** represents p < 0.001, and n.s. represents p > 0.05.

As shown in **Figures 2A, B**, *A. sobria* infection led to ASC oligomerization and caspase-1 p20 production, which showed that *A. sobria* triggered ASC-dependent inflammasome activation in PMφs. To explore the roles of ASC-dependent inflammasome in pro-inflammatory cytokine of IL-1β secretion, inhibition assays were conducted. Caspase-1 specific inhibitor of Ac-YVAD-CHO or pan-caspase inhibitor of zVAD-fmk were individually inoculated into PMφs, and results showed that these two inhibitors successfully decreased p20 expression. Moreover, zVAD-fmk could thoroughly inhibit p20 generation in comparison with Ac-YVAD-CHO. They had no effects on the expression of pro-IL-1β protein, but significantly lowered the oligomerization of ASC and secretion of IL-1β with similar trend in p20 generation (**Figure 2C**). Overall, these data illustrated that *A. sobria*–mediated IL-1β p17 secretion is regulated by ASC-dependent inflammasome.

### NLRP3 Inflammasome Activation in Response to *A. sobria* Stimulation

**Figure 3:**
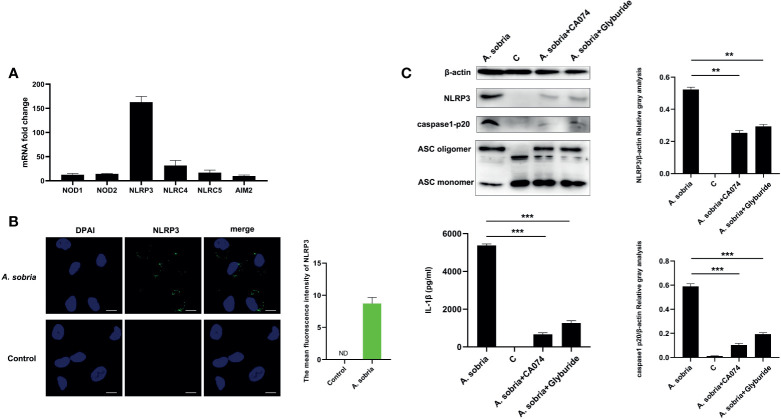
NLRP3 inflammasome activation in response to *A sobria* stimulation and block NLRP3 inflammasome downregulated IL-1β secretion. PMφs (4.5 × 10^6^ cells/well in six-well plate) were infected with *A sobria* (MOI = 10) for 90 min, and then *A sobria* was discarded. After washing three times with sterile PBS, PMφs were treated with gentamicin sulfate (100 μg/ml) containing RPMI 1640 maintenance medium for 2 h to kill residual *A sobria* and then replaced with gentamicin sulfate (20 μg/ml) containing RPMI 1640 maintenance medium for 12 h **(A)** Total RNA was extracted from infected cells, and mRNA levels of NOD1, NOD2, NLRP3, NLRC4, NLRC5, and AIM2 were measured using qPCR. **(B)** Location of NLRP3 protein was observed using immunofluorescence. The green signals were FTIC-labeled NLRP3 protein, and the blue signals were DAPI-stained cell nucleus. Bar = 10 μm. The mean fluorescence intensity of NLRP3 was measured using Fiji software, and graph was generated from two dependent assays. **(C)** PMφs were pretreated with either CA-074 methyl ester (25 μM) or Glyburide (50 μM) for 1 h prior to infection. The protein expression levels of NLRP3 in the cells, caspase-1 p20 levels in the supernatants, and the ASC oligomerization levels in the cross-linked cell pellets were detected using western blotting. Secretion of mature IL-1β were measured using ELISA. The relative gray values of NLRP3 and caspase-1 p20 were normalized to β-actin through Image. Data were presented as mean ± Standard Deviation from three independent assays with three technical repeats. Significant difference was analyzed using SPSS software, and graph was generated using GraphPad Prism 8 software. ** represents p < 0.01 and *** represents p < 0.001.

Common PRRs of NOD1, NOD2, NLRP3, NLRC4, NLRC5, and AIM2 were detected using qPCR assays after 90 min of infection with *A. sobria* at a MOI of 10 and 2 h gentamycin treatment followed by 12 h incubation. NLRP3 was the highest activated receptor by calculating the mRNA expression levels (**Figure 3A**). Location analysis of NLRP3 receptor using mouse NLRP3 mAbs showed that NLRP3 displayed puncta-like surrounding nuclear after infection with *A. sobria*, and in contrast, no NLRP3 signals were detected in the unstimulated cells (**Figures 3B** and **S2**). These data indicated that NLRP3 inflammasome was activated in response to *A. sobria* in PMφs. To determine the roles of NLRP3 receptor, inhibitors of CA-074 methyl ester or Glyburide were inoculated into PMφs for 1 h prior to *A. sobria* infection, and NLRP3 protein expression was significantly inhibited to some degree. Meanwhile, these two inhibitors decreased ASC oligomerization levels in comparison with that in the *A. sobria* infection group. After blocking of NLRP3 inflammasome through downregulation of NLRP3 protein and inhibition of ASC oligomerization, the activated caspase-1 p20 was also obviously inhibited, with accompanying dramatic decrease in the secretion of IL-1β. These data indicated that inhibition of *A. sobria*–induced NLRP3 inflammasome production restricted caspase-1 p20 activation, then reduced IL-1β secretion (**Figure 3C**).

The NLRP3 inflammasome in PMφs induced by *A. sobria* was monitored from 6 to 24 h after inoculation. The results showed that *A. sobria* could lead to caspase-1 p20 activation as early as 6 h of inoculation; the production amounts slightly increased when measured at 12 h and dramatically upregulated to the peak at 24 h. A similar trend appeared in the generation of IL-1β (**Figure S3**). These data illustrated that *A. sobria* induced PMφs NLRP3 inflammasome activation in a time-dependent manner.

### Inhibition of NLRP3 Inflammasome Triggerred IL-1β Secretion Weakens *A. sobria*–Induced Inflammation in PMφs

**Figure 4:**
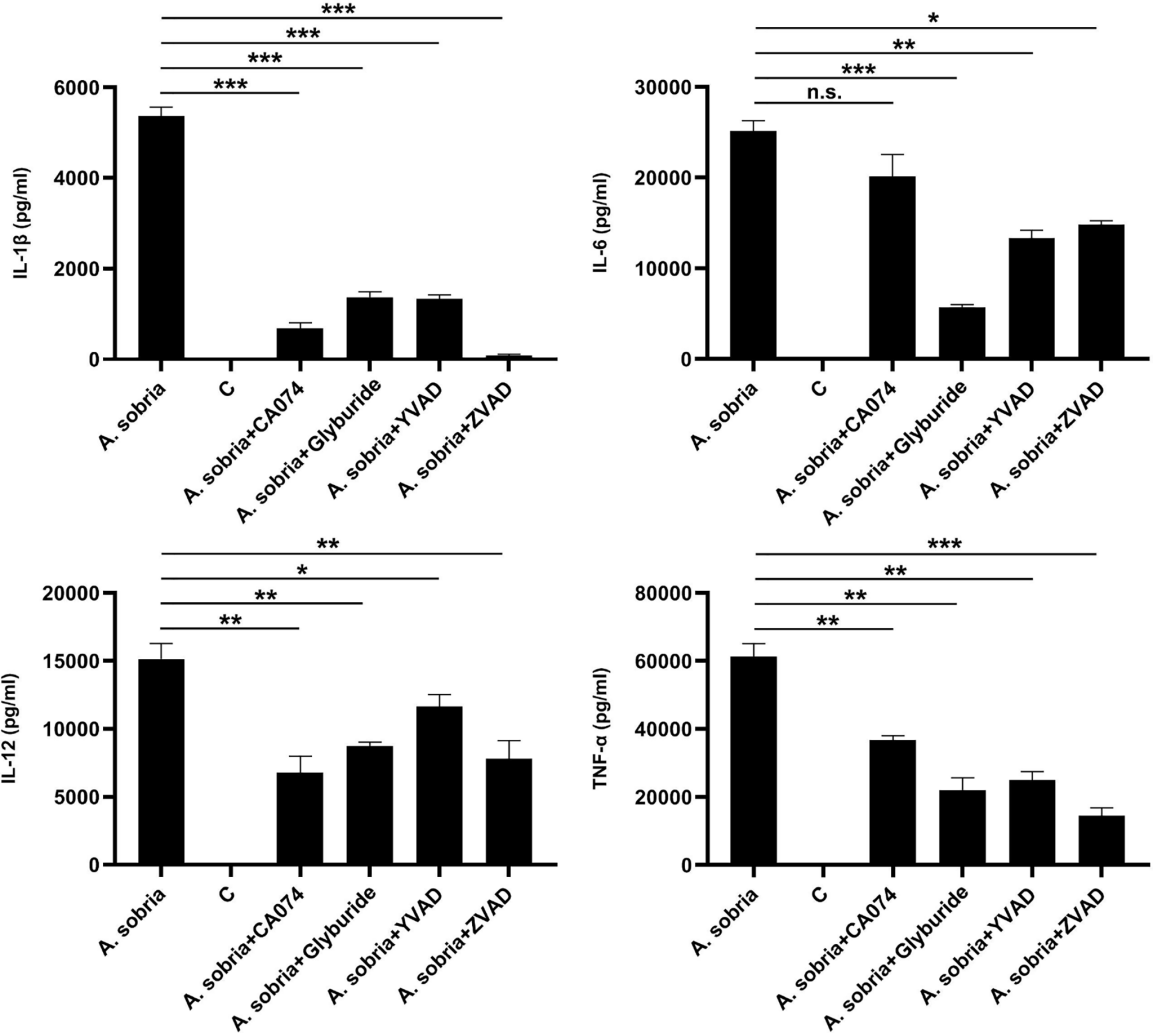
Downregulation of IL-1β secretion restricted *A. sobria*–induced pro-inflammatory cytokine secretion in PMφs. PMφs (4.5 × 10^6^ cells/well in six-well plate) were pretreated with CA-074 methyl ester (25 μM), Glyburide (50 μM), Ac-YVAD-CHO (100 μM), or zVAD-fmk (10 μM) for 1 h, infected with *A. sobria* (MOI= 10) for 90 min, and then *A. sobria* was discarded. After washing three times with sterile PBS, PMφs were treated with gentamicin sulfate (100 μg/ml) containing RPMI 1640 maintenance medium for 2 h to kill residual *A. sobria* and then replaced with gentamicin sulfate (20 μg/ml) containing RPMI 1640 maintenance medium for 12 h. No inhibitor-treated groups were used as positive control group, and no *A. sobria* infection group was used as negative control (C) group. The supernatants were collected for proinflammatory cytokines of IL-6, IL-12, and TNF-α measurement using ELISA. Data were presented as mean ± Standard Deviation from three independent assays with three technical repeats. Significant differences between positive control group and inhibitor treatment groups were analyzed using SPSS software, and graph was generated using GraphPad Prism 8 software. * represents p<0.05, ** represents p < 0.01, *** represents p < 0.001, and n.s. represents p > 0.05.

The influence of these four kinds of *A. sobria*–induced NLRP3 inflammasome inhibitors on other pro-inflammatory cytokines was determined through ELISA assays. The results showed that blocking of NLRP3 inflammasome activation significantly downregulated the secretion of IL-1β and obviously reduced the secretion of IL-6, IL-12, and TNF-α, except for IL-6 levels after pretreatment with inhibitor of CA-074 methyl ester (**Figure 4**). Overall, these data revealed that blocking the production of NLRP3 inflammasome could fight against *A. sobria*–induced inflammation *in vitro*.

## Discussion

*Aeromonas* are emerging pathogenic microorganisms to humans especially for immunocompromised or immunocompetent patients in underdeveloped nations (Janda and Abbott, 2010). It is estimated that the infection rate of *Aeromonas* ranges from 1.5 to 76 cases per million individuals with clinical symptoms of gastroenteritis, septicemia, or wound infections (Lamy et al., 2009; Wu et al., 2014). Among them, bacteremia caused by *A. sobria* lead to the highest mortality rate in *Aeromonas* (Janda and Brenden, 1987; Martins et al., 2002). After pathogen invasion, the innate immune system of hosts is activated, and PRRs on the surface of cell membranes or within cells behave as detectors to recognize the exogenous PAMPs or its released DAMPs. Previous studies on *A. sobria* were mainly focused on the case reports in humans, and several studies were about the characteristics and functions of virulence factor serine protease in colonic epithelial cells (Kobayashi, 2011; Kobayashi et al., 2019; Song et al., 2019). However, the interactions between *A. sobria* and host cells’ innate immunity have not been clarified. Thus, the present study explored the effects of *A. sobria* on the host cell innate immunity using primary mouse peritoneal macrophages and its potential mechanisms.

Previous studies showed that mouse intestinal tract produced Th1 cytokine response after *Aeromonas caviae* infection; Nile tilapia released interleukins to defend infection of *Aeromonas hydrophila*; and milkfish liver produced inflammation response after *Aeromonas hydrophila* invasion (Hayes et al., 2010; Ragasa et al., 2019; Zhang et al., 2019). Thus, these inflammation-related cytokines were essential for organisms to eliminate and control *Aeromonas* infection in humans or animals. Primary macrophages are of good choice for exploring the immune inflammatory response mechanisms induced by pathogenic microorganism. Researchers explored the *Aeromonas salmonicida* subsp. *Salmonicida*–induced immune response using cod primary macrophages (Soto-Dávila et al., 2019). Previous studies showed that the mRNA expression of M1/M2-like macrophage markers was higher in PMφs than bone marrow–derived macrophages isolated from female C57BL/6 mice (Bisgaard et al., 2016). M1 macrophages have pro-inflammatory effects to fight against bacterial infection, while M2 macrophages are involved in tissue remodeling and fibrosis (Benoit et al., 2008; Butcher and Galkina, 2012). Thus, primary PMφs from mouse were used to establish the cellular infection models to study the inflammatory response mechanisms in host cells triggered by *A. sobria* in our study. Various pro-inflammatory cytokines were measured after infection of *A. sobria* in PMφs. Similar to other *Aeromonas* microorganism, the mRNA levels of many cytokines were upregulated and IL-1β increased to the highest levels. Moreover, the generated protein levels of IL-1β were in a dose-dependent manner when infection with MOIs ranged from 1 to 100.

Mature IL-1β was regulated by two signals; the nuclear transcription factor of NF-κB mediated generation of pro-IL-1β and then activated caspase cleaved pro-IL-1β into mature IL-1β p17 releasing into extracellular (Perkins, 2006; Man et al., 2017). Thus, caspase-1 p20 was detected in the supernatants released by *A. sobria*–infected PMφs, and caspase-1 was activated as expected after *A. sobria* invasion. Moreover, the roles of caspase-1 p20 were determined through inhibition assays, and results showed that the release of mature IL-1β p17 was dependent not only on the cleavage of active caspase-1 but also on caspase-11. In addition, *A. sobria* invasion triggered ASC oligomerization. These data illustrated that *A. sobria* infection led to ASC-dependent inflammasome activation. Many studies indicated that activation of caspase-1 was a marker for canonical inflammasome complex assembling (Man and Kanneganti, 2015). The intracellular NOD-like receptors play important roles in the mechanisms of host defense and the pathogenesis of inflammatory diseases (Kanneganti et al., 2007; Zaki et al., 2012). Thus, the transcription levels of some common receptors were first screened through qPCR assays, and the results revealed that NLRP3 receptor showed the highest upregulated level. NLRP3 detects a broad range of microbial motifs, endogenous danger signals, and environmental irritants, resulting in the activation of NLRP3 inflammasome (Swanson et al., 2019). Location analysis showed that *A. sobria* infection made host NLRP3 protein displayed puncta-like surrounding nuclear in PMφs. These data fully confirmed that NLRP3 inflammasome was activated in PMφs during *A. sobria* infection. During this process, NLRC4 and NLRC5 receptors were also upregulated to higher levels than other receptors. It is reported that NLRC5 plays roles in the regulation of major histocompatibility complex (MHC) class I and II antigen presentation (Meissner et al., 2010; Zuo and Rowe, 2012). Moreover, NLRC5 deficiency does not influence cytokine induction by virus and bacterial infections (Kumar et al., 2011). Thus, we dismissed the NLRC5 receptor for the following research. NLRC4 responds to bacterial flagellin and a conserved type III secretion system (TTSS) rod component, recruits pro-caspase-1 to directly form NLRC4 inflammasome and involves in a variety of pathogenic bacterial infection, such as *Salmonella*, *Shigella*, *Pseudomonas aeruginosa* (Zhao et al., 2011; Kesavardhana and Kanneganti, 2017; Duncan and Canna, 2018). The roles of NLRC4 in *A. sobria* infection should be further studied in the future.

Formation of NLRP3 inflammasome was attributed to many factors, such as K^+^ efflux, ROS generation, cathepsin B ways, etc. (He et al., 2016). In this study, inhibitors of Glyburide (block K^+^ efflux) (Mohamed et al., 2009) and CA-074 methyl ester (block cathepsin B) were used to explore its effects on the activation of caspase-1 and mature of IL-1β (Wang et al., 2017; Wei et al., 2020; Zhao et al., 2021). The results showed that these two inhibitors blocked NLRP3 inflammasome through decreasing NLRP3 protein expression levels and ASC oligomerization degree. Moreover, blocking NLRP3 inflammasome not only inhibited caspase-1 p20 expression levels but also downregulated IL-1β p17 secretion. These data illustrated that *A. sobria*–triggered activation of NLRP3 inflammasome in PMφs was closely related to K^+^ efflux and lysosomal damage ways. Previous studies showed that *A. hydrophila*–triggered NLRP3 inflammasome activation in macrophages was mediated by its produced cytotoxins, including aerolysin, hemolysin, and multifunctional repeat-in-toxin. For *A. sobria*, it could also produce cytotoxins, and hemolysin-mediated $\text{HC}{\text{O}}_{3}^{-}$ secretion was correlated with the severity of inflammatory diarrhea (Takahashi et al., 2006). Whether hemolysin is associated with the NLRP3 inflammasome activation in *A. sobria*–stimulated PMφs needs to be further explored. *A. hydrophila* could induce apoptosis in a variety of cells, such as head kidney macrophages, murine macrophages, human intestinal epithelial cells, etc. (Galindo et al., 2004; Banerjee et al., 2014). NLRP3 inflammasome activation will probably lead to pyroptosis, a kind of cell death, and contributes to the pathogenesis of many diseases (Wree et al., 2014; Qiu et al., 2017; Zeng et al., 2020). Our study preliminarily found that *A. sobria* induced cell pyroptosis by releasing LDH, while the underlying mechanisms and its roles in *A. sobria* disease development needs to be further explored in the future.

IL-1β was regarded as an important marker in various inflammatory diseases, such as inflammatory bowel diseases, neuroinflammatory diseases, periodontitis, etc. (Mao et al., 2018; Mendiola and Cardona, 2018; Cheng et al., 2020). Inhibitors of Glyburide, CA-074, Ac-YVAD-CHO, or zVAD-fmk were used to restrict the secretion of IL-1β, and then inflammatory cytokines of IL-6, IL-12, and TNF-α were measured (Mohamed et al., 2009; Wang et al., 2017; Sollberger et al., 2018; Wang et al., 2018a; Wang et al., 2018b; Zhao et al., 2021). The results showed that downregulation of IL-1β could resist *A. sobria*–induced inflammatory response in PMφs. These data imply that IL-1β may be a promising therapeutic target for the treatment of *A. sobria* infection.

In conclusion, we established an *A. sobria* infection model *in vitro* using primary mouse peritoneal macrophages, aiming to explore the host cell defense mechanisms against *A. sobria* invasion. Our findings indicated that *A. sobria* infection triggered death and pro-inflammatory cytokine production in PMφs through activating NLRP3 inflammasome, and activation of caspase-1-mediated IL-1β p17 secretion plays vital roles in the inflammatory response. These findings may provide reference for elucidating the pathogenic mechanism of *A. sobria in vivo* in future studies.

## Data Availability Statement

The original contributions presented in the study are included in the article/**Supplementary Material**. Further inquiries can be directed to the corresponding authors.

## Ethics Statement

The animal study was reviewed and approved by the Animal Welfare and Research Ethics Committee of Jiangsu Ocean University (Permit Number: 2017124242).

## Author Contributions

JD and PZ: Designed the research. WZ, ZL, HY, and GW: Conducted the research. ZL, HY, GL, and WY: Analyzed the data. WZ: Wrote the manuscript. BB edited the English language. JD and WL: Directed the project. All authors contributed to the article and approved the submitted version.

## Funding

The authors thank the Natural Science Foundation of Jiangsu Province (BK20191210), the Open Foundation of Key Jiangsu Institute of Marine Resources Development (JSIMR202016), the Jiangsu Distinguished Professor program (KK19515), the fifth phase of “333 Project” scientific research project in Jiangsu Province (BRA2019248), the Subject of Lianyungang Science and Technology Bureau (SF2015), and the Priority Academic Program Development of Jiangsu Higher Education Institutions of China for financial support. They had no role in study design, data collection and analysis, decision to publish, or preparation of the manuscript.

## Conflict of Interest

The authors declare that the research was conducted in the absence of any commercial or financial relationships that could be construed as a potential conflict of interest.

## Publisher’s Note

All claims expressed in this article are solely those of the authors and do not necessarily represent those of their affiliated organizations, or those of the publisher, the editors and the reviewers. Any product that may be evaluated in this article, or claim that may be made by its manufacturer, is not guaranteed or endorsed by the publisher.
