# Supplementary material for: Aeromonas sobria Induces Proinflammatory Cytokines Production in Mouse Macrophages via Activating NLRP3 Inflammasome Signaling Pathways
Source: Front Cell Infect Microbiol. 2021 Aug 26;11:691445. doi: 10.3389/fcimb.2021.691445 (PMC8428973; doi:10.3389/fcimb.2021.691445)
Supplement: Supplementary file 2 [file DataSheet_2.docx]

**Supplementary FIGURE 1** The mRNA fold change of IL-1β and IL-6 in *A. sobria* infected PMφs (MOI= 10) with 20-fold diluted cDNA and 40-fold diluted cDNA as templates for qPCR assays.

**
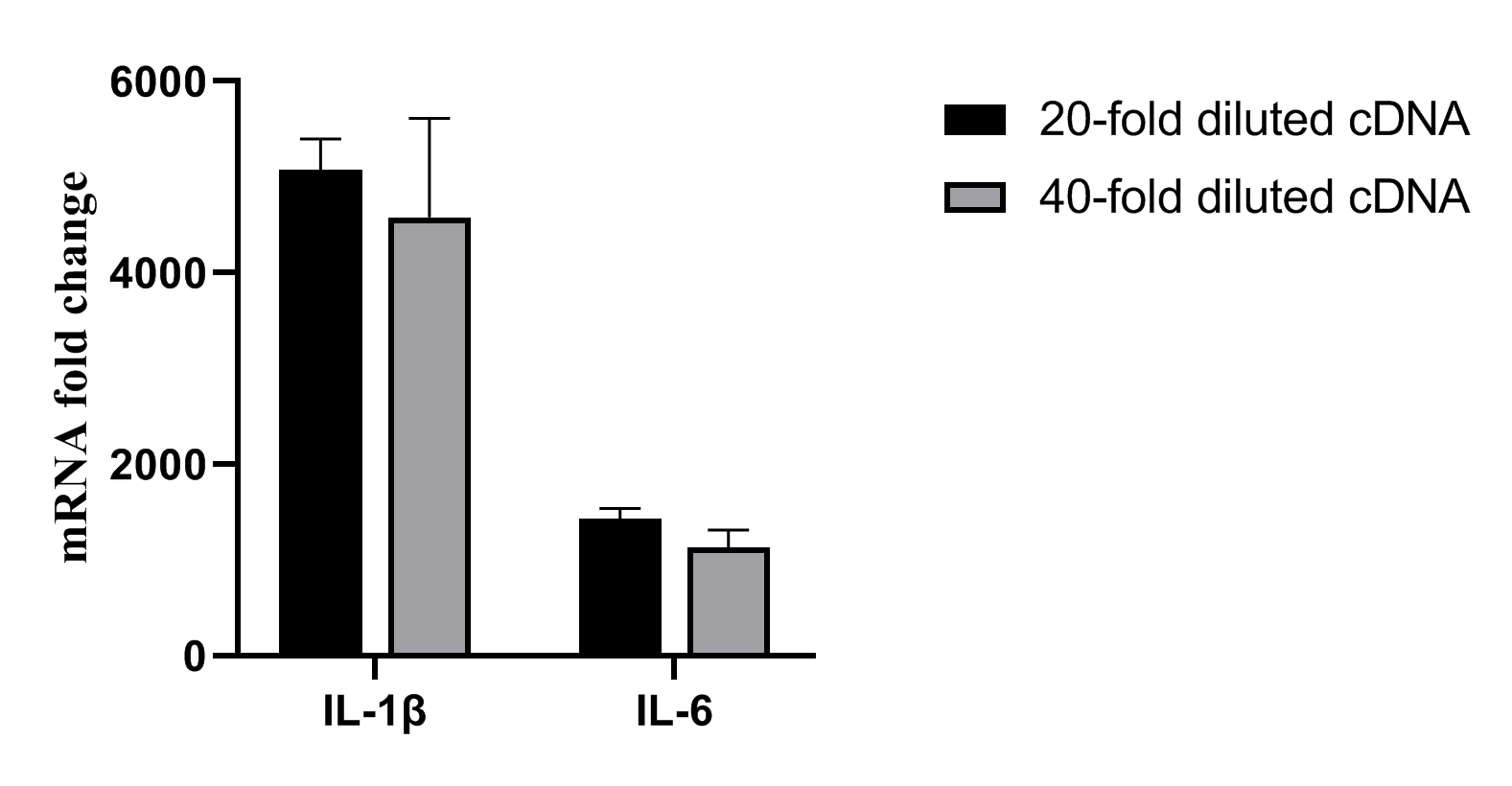
**

**Supplementary FIGURE 2** Immunofluorescence observation of NLRP3 protein location. PMφs (4.5 × 10^6^ cells/well in 6-well plate) were infected with *A. sobria* (MOI= 10) for 90 min and then *A. sobria* was discarded. After washing three times with sterile PBS, PMφs were treated with gentamicin sulfate (100 μg/mL) containing RPMI 1640 maintenance medium for 2 h to kill residual *A. sobria* and then replaced with gentamicin sulfate (20 μg/mL) containing RPMI 1640 maintenance medium for 12 h. The infected PMφs were used for immunofluorescence analysis of the location of NLRP3 protein. The green signals were FTIC-labeled NLRP3 protein and the blue signals were DAPI-stained cell nucleus. Bar =10 μm.

**FIGURE S2**

**
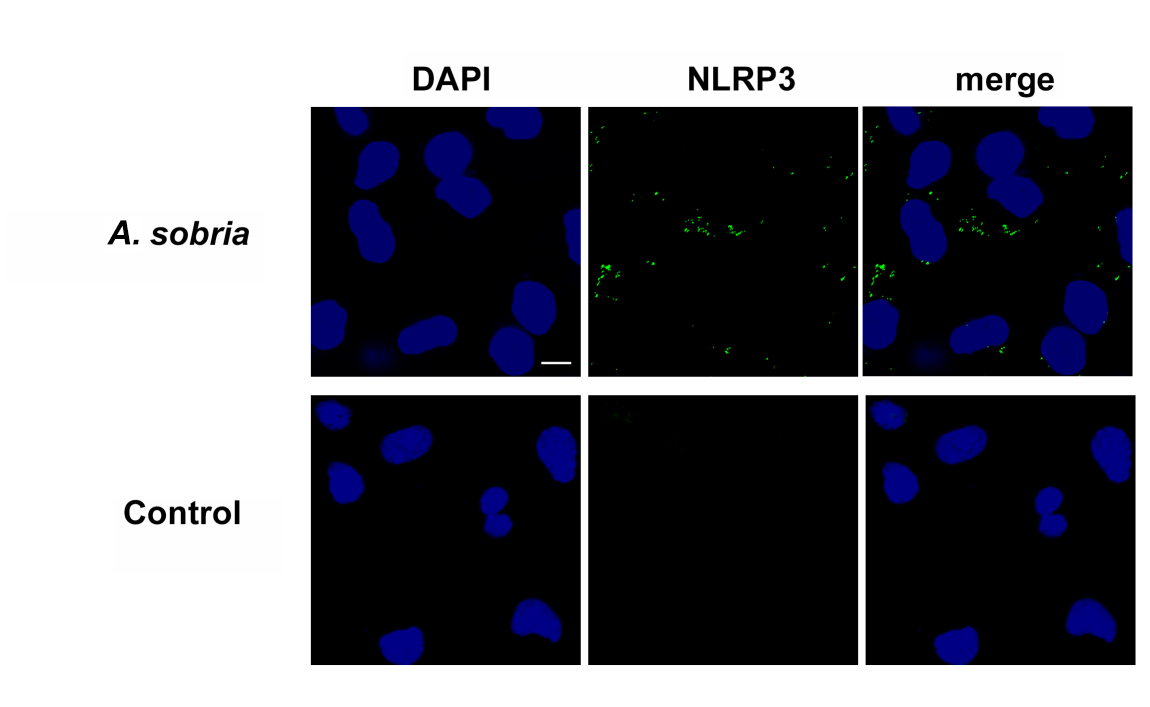
**

**Supplementary FIGURE 3** *A. sobria* triggered PMφs NLRP3 inflammasome activation in a time-dependent manner. PMφs (4.5 × 10^6^ cells/well in 6-well plate) were infected with *A. sobria* (MOI= 1) for 90 min and then *A. sobria* was discarded. After washing three times with sterile PBS, PMφ were treated with gentamicin sulfate (100 μg/mL) containing RPMI 1640 maintenance medium for 2 h to kill residual *A. sobria* and then replaced with gentamicin sulfate (20 μg/mL) containing RPMI 1640 maintenance medium for 6 h, 12 h, 24 h, respectively. Protein was extracted from infected cells and concentrated from harvest supernatants. The protein expression of caspase-1 p20 in the supernatants was detected using western blotting. The relative gray values of caspase-1 p20 was normalized to β-actin through Image J. Data were presented as mean±Standard Deviation from three independent assays with three technical repeats. Significant difference was analyzed using SPSS software and graph was generated using GraphPad Prism 8 software. * represents p<0.05, ** represents p<0.01 and*** represents p<0.001.

**FIGURE S3**

**
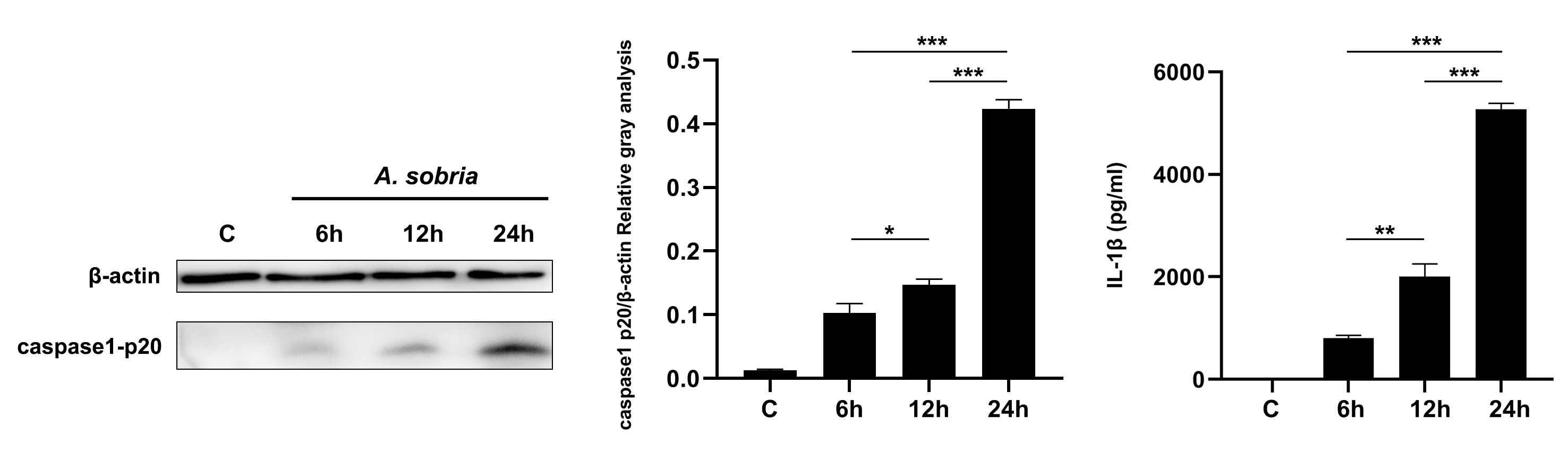
**
